# Feathered Lectures—Evidence of Perceptual Factors on Social Learning in Kea Parrots (*Nestor notabilis*)

**DOI:** 10.3390/ani14111651

**Published:** 2024-05-31

**Authors:** Lucie Marie Gudenus, Amelia Wein, Remco Folkertsma, Raoul Schwing

**Affiliations:** 1 MeiCogSci, Middle European Interdisciplinary Master’s in Cognitive Science, University of Vienna, 1010 Vienna, Austria; 2 Comparative Cognition, Messerli Research Institute, University of Veterinary Medicine Vienna, 1210 Vienna, Austriaraoul.schwing@vetmeduni.ac.at (R.S.)

**Keywords:** stimulus enhancement, parrot cognition, avian cognition, parrot behavior

## Abstract

**Simple Summary:**

In this study, we investigated whether Kea parrots (*Nestor notabilis*) can learn to solve a task to reach a food reward by watching another Kea solve the task first, a process known as social learning. The task that the Kea needed to solve was a box with two strings attached, where pulling the correct string caused a reward to be released into a food tray. Pulling the incorrect string had no effect. Kea subjects were separated into groups: test and control. ‘Test subjects’ observed an experienced, trained demonstrator first solve the box, after which they were given access to the box to try and solve for themselves. ‘Control subjects’ did not view a demonstration and were given access to the box with no previous experience. We could show that test subjects spent significantly more time manipulating the correct string compared to control subjects. This suggests that viewing a demonstration brought the test subjects’ attention to the relevant parts of the box. There was also a trend towards test subjects having more success in solving the box compared to control subjects.

**Abstract:**

Social learning describes the acquisition of knowledge through observation of other individuals, and it is fundamental for the development of culture and traditions within human groups. Although previous studies suggest that Kea (*Nestor notabilis*) benefit from social learning, experimental evidence has been inconclusive, as in a recent two-action task, all perceptual factors were ignored. The present study attempts to address this by investigating social learning in Kea with a focus on social enhancement processes. In an experiment with a captive group of Kea, we investigated whether individuals that had the opportunity to observe a conspecific performing a simple task subsequently show better performance in that task than a control group without prior demonstration. This study provides a strong tendency of greater success in skill acquisition in Kea as a result of social learning. Kea that observed a conspecific solving a task showed clear evidence of perceptual factors drawing attention to the relevant parts of the experimental apparatus and manipulated these significantly more (100% of trials) than control birds (77.8% of trials). Combined with a strong trend (*p* = 0.056) of the test subjects solving the task more than the control subjects, this shows conclusively that Kea, at least when required to solve a task, do attend to perceptual factors of a demonstrated action.

## 1. Introduction

In the animal kingdom, social influences on adaptive behavior can be found in a variety of taxa, including insects [1], birds [2], reptiles [3], fish [4], rodents [5], cetaceans [6], and primates [7,8], and in domains such as food preferences, hunting and foraging techniques, tool use, habitat choice, mate choice, predator recognition and song learning (e.g., [9,10,11,12] for reviews). Social learning has been at the core of the debate over possible culture in animals for decades [13,14], as this accumulation and transfer of knowledge between individuals is at the core of human civilization. While direct comparison depends strongly on the specific definition of culture, social learning will always be a fundamental requirement, and it is thus of great interest to the field of animal cognition, but also biology in general, to investigate this type of information transfer in all of its facets.

Social learning itself can be facilitated by different mechanisms [10,12,15,16,17]. Most established classification schemes comprise the same main mechanisms of social learning: copying, which can be further distinguished into imitative and emulative copying behavior, observational conditioning, and enhancement [18,19]. However, these categories are partially overlapping and not hierarchical, as, e.g., experimental investigation of the milk-bottle opening by Blue Tits (*Cyanistes caeruleus*) showed evidence of not only the previously described local enhancement (attending to the location of the lid), but observational learning effects as well [20] (associating the outcome of access to the milk bottle with the interaction of the demonstrator).

Enhancement processes, based on perceptual factors (e.g., color or location of an object, or part thereof, being manipulated), are attentional mechanisms that give information about the orientation towards a stimulus [12]. The observer adopts the same focus as the demonstrator and subsequently acts similarly either by chance, by acting out species-typical behavioral patterns, or based on the affordances of the object, i.e., information about the possibilities to interact with said object. Enhancement can be further subdivided into stimulus and local enhancement [17,19,21,22,23]. When an individual observes a demonstrator operate in a specific location, it is more likely that the observer then interacts with objects at this location, which is called local enhancement [22]. When the observer’s attention is drawn to an object or part of an object, regardless of the location, it is called stimulus enhancement [17].

Social learning in birds has been extensively studied, as it is integral to song learning in vocal learners, e.g., passerines [24]. However, with regard to physical cognition, where actions are demonstrated, the number of studies is significantly lower. Nonetheless, several bird species have shown themselves capable of learning socially from conspecifics, e.g., Japanese Quail (*Coturnix japonica*) and European Starlings (*Sturnus vulgaris*) [25,26]. In Common Ravens (*Corvus corax*) [27], sibling subjects showed stimulus enhancement effects when observing a conspecific demonstrator manipulating even an unrewarded object. In psittaciform, budgerigars (*Melopsittacus undulates*) have been shown to learn socially from conspecifics [28,29]. In Goffin’s Cockatoos (*Cacatua goffini*) [30], demonstrations of tool use resulted in the transfer of tool-use competence to subjects in the observer group, but not the ghost control group.

Kea (*Nestor notabilis*) are large omnivorous parrots that are endemic to the alpine areas of New Zealand [31]. Kea are known for their problem-solving skills [32,33], flexibility [34,35], neophilic–explorative behavior [36], as well as their playfulness [37]. They also have several characteristics that would make them prime candidates for social learning. Their natural curiosity, gregariousness, social tolerance, prolonged juvenile phase and long life span [31] are all factors that would facilitate social learning [38]. Additionally, the large documented number of potential food sources exploited by Kea in their natural environment [39,40] strongly suggests a transfer of information within the social group.

Juvenile individuals learn about the social structures of the flock, and pay attention and learn from the actions of their conspecifics as well as the objects with which they are interacting [31]. Therefore, it can be theorized that cues about affordances of the environment and objects could be obtained from other conspecifics. The importance of social information for problem solving has been experimentally studied in groups of captive and wild Kea [33,35,38,41,42]. Although the results point to the use of social information in Kea, results from past studies have been mixed regarding if perceptual information is used by Kea.

A recent study with Kea [43] aimed to replicate a two-action task first performed with budgerigars [44]. Here, subjects in the observer group watched a demonstrator push one of two differently colored foam disks, randomized in left-right position, into the box, to gain access to a reward. While a social learning effect, in the form of a faster initial solving rate for the observer group over the control group, was found, the mode of solving (push vs. pull), as well as perceptual factors (color and position of disk) were not copied by the observers. As previous studies utilizing objects/tools [35,41] or action sequences [42] did show perceptual factors (stimulus enhancement and local enhancement, [12]) being attended to in social learning, the question arose as to why the Kea had not done so in the two-action task. One possible explanation was that the simplicity of the task, but also the fact that both actions, both colors, and/or both positions were actually rewarded, was not limiting enough to require the Kea to abandon their overtly explorative nature. Therefore, we decided to follow up with a setup where perceptual factors were required to differentiate between the correct choice, as demonstrated by a conspecific, and the incorrect choice, to address if kea, unlike other birds, do not pay attention to such aspects of a demonstration.

The task in question was a box that could be solved by pulling a string, which released a piece of peanut into a reward tray. There were two differently colored strings attached to the box—one functional and one non-functional. Naïve observers watched a knowledgeable demonstrator solve the task, after which they gained access to the box. The behavior of the observers was compared to that of a control group, which received no demonstration. The analysis focused on success in solving the task, as well as indicators of perceptual factors. Based on the reviewed literature, the following research questions were posed. (1) Does observing a knowledgeable demonstrator solve a task increase the success of naïve observers compared to non-observer controls? This would support the idea that Kea use some aspect of social learning to solve a task. We hypothesized that observing a demonstrator solve a task would affect the actions of observer vs. non-observer subjects. Based on the proposed apparatus, we predicted that more of the observer subjects would successfully solve the task and obtain the reward when compared to subjects from the control group without a demonstration. (2) Is there evidence that naïve observers are more likely to manipulate the correct string following their observation of a demonstration, compared to non-observer controls? This would support perceptual factors as being integral in Kea social learning. Here, we hypothesized that observing a demonstrator manipulate one of two strings, distinguishable by color, to solve a task, would affect the actions of observer vs. non-observer subjects. Here, we predicted that observers would spend more time manipulating the demonstrated string when compared to subjects from the control group without a demonstration.

## 2. Materials and Methods

### 2.1. Subjects and Housing

**Figure 1:**
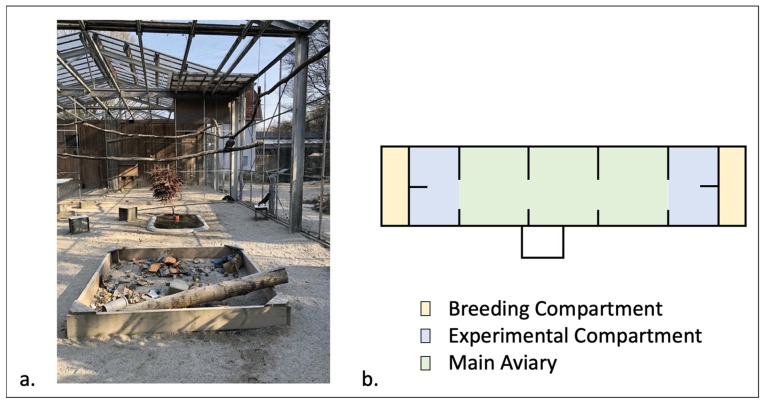
Kea aviary at Haidlhof Research Station. (**a**) Main aviary with hanging branches, ponds, shelter structures and feeding tables. (**b**) Schematic plan of the aviary.

The Kea were tested at the Kea Lab, located at the Haidlhof Research Station in Bad Vöslau, Austria. This captive group consisted of 26 Kea that are permanently housed in a large outdoor aviary (52 m (W) × 10 m (L) × 4 m (H), Figure 1). The youngest birds available to participate were one year old at the time of study (hatched 2022), and the oldest was 23 at the time of study (hatched 1999).

The aviary was equipped with sand substrate, perches, shelters, feeding tables, and environmental enrichment, which was regularly renewed. Feeding took place three times daily (9:00, 12:00, 15:30) and consisted of a mixture of fruit, vegetables, seeds, and a protein source once daily (cooked meat or eggs). Water was available ad libitum in water dishes and small ponds. After the experiment was concluded, all birds stayed at the Haidlhof Research Station.

The Kea were never food-deprived for the purpose of experiments, and only positive reinforcement was used. The rewards offered to the Kea in experimental settings were not part of the normal diet, but were treats that they could only obtain during experiments. Previous food-preference tests have shown that Kea do indeed prefer these rewards over food items from their normal diet [45]. In this study, the subjects received one half of a peanut seed per trial as a reward.

Testing took place between March and June 2022, and a second round of testing took place in June 2023. Altogether, 24 Kea participated in this study (see Table 1 and Supplementary Materials Table S2 on subjects’ previous experience). Subjects were tested two times daily, three days per week: once in the morning (10:00–12:00) and once in the afternoon (13:30–15:30). A session consisted of one two-minute observation period where the subject was allowed to either view a demonstration (test group) or view the test apparatus (control group). This was followed immediately by the subject being given access to the test apparatus for two minutes to try and solve the task (more detailed information is provided in the appropriate sections below).

There were experimental compartments located on each end of the aviary, which could be closed off from the rest of the aviary. Outside testing times, the experimental compartments remained open to function as part of the main living area of the Kea. The Kea were trained to enter and exit the different compartments on verbal command. All subjects participated voluntarily in the experiments by following the researcher’s commands to enter the experimental compartment. The birds then either walked, flew, or were carried on the experimenter’s arm into the testing area. They were free to ignore the commands if they were not motivated to participate in the tests. The Kea could end an experimental session at any time by retreating to hanging branches or shelter structures, or in any other way refusing to participate. If a subject ended a session early, it was sent back into the main aviary by verbal command. If any subject refused to participate three times in a row, it was excluded from the experiment.

The subjects were separated into four groups: Test Group 1 (TG1), Test Group 2 (TG2), Control Group 1 (CG1) and Control Group 2 (CG2). The first subjects tested were CG1, and after that phase was complete, two of the adult males from that group (An, Sk) were then re-trained to act as demonstrators for the two test groups. We trained two demonstrators instead of just one because we know from previous experiments that demonstrators can become demotivated if they are required to demonstrate too often [43]. It was also not possible to train more than two demonstrators due to the limited number of subjects in the study. Each test subject always observed the same demonstrator, creating the two test groups. We attempted to balance all groups for age, sex, and dominance rank.

The initial data collection occurred during the breeding season of 2022 (February–April at the Haidlhof Research Station), which limited the number of subjects that could participate to those that were not involved in breeding. Adult females become highly territorial and aggressive towards other Kea besides their partners during the breeding season [46], and generally refuse to participate in any form of testing. When this occurs, they must be separated from the group to reduce aggression and to be able to live out their breeding behavior [47,48]. One subject, Ma, was an adult female, but she was an exception as she was unpaired and not showing aggression towards the other birds. We therefore attempted to include her in the study in 2022, but she was not motivated to work, so she was excluded. All other birds that began the study also went on to complete it, and no bird except Ma showed lack of motivation to participate in any trial. The birds that did not participate in 2022 due to breeding, or because they were too young, took part in the second control group in June 2023.

### 2.2. Experimental Setup

**Figure 2:**
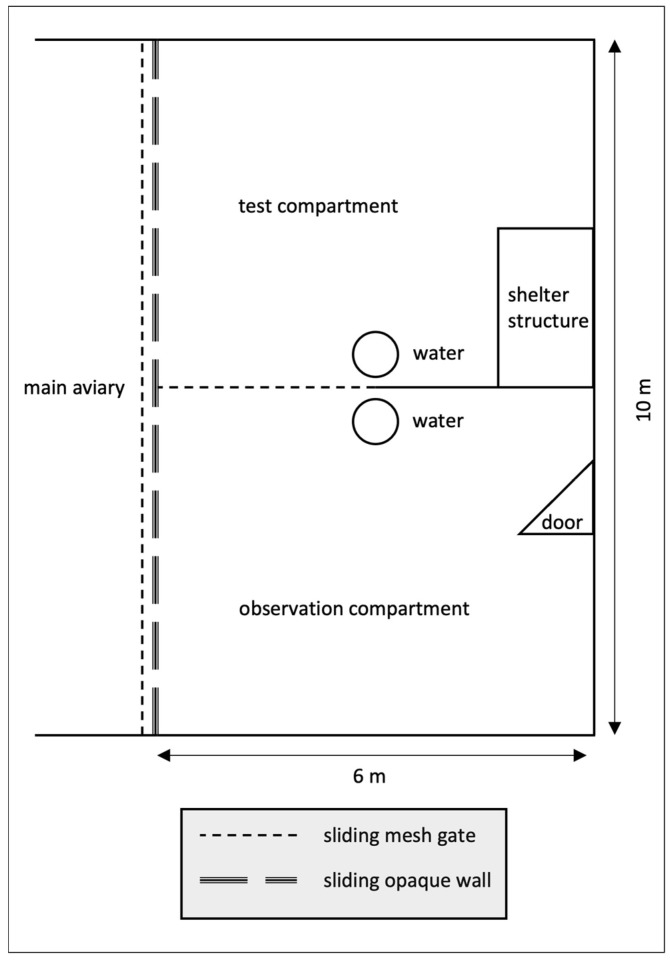
Schematic of experimental compartment.

All stages of the experiment took place in the experimental compartments (10 m (W) × 6 m (L) × 4 m (H), Figure 2). These compartments could be closed off from the main aviary with a sliding wire-mesh door and visually occluded with sliding opaque white walls, which remained closed during testing to separate the subjects from the rest of the group. Each experimental compartment could be further divided into equally sized sub-compartments—an observation compartment and a test compartment—with a sliding wire-mesh gate (Figure 2).

### 2.3. Apparatus

**Figure 3:**
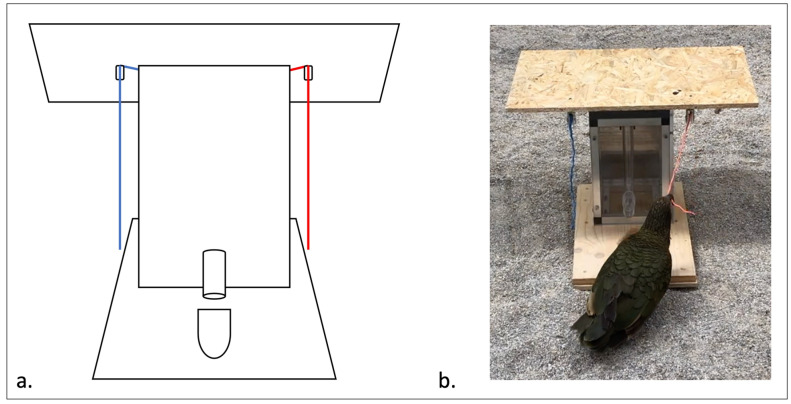
Test apparatus. (**a**) Schematic of apparatus with two differently colored strings on each side of the test box and a tube that released the reward into the feeding tray in front of it. (**b**) Kea manipulating the test box. The board on top was necessary to prevent the Kea from manipulating the top of the box or the strings from a position other than standing on the ground.

The apparatus used in both the controls and social learning tests was a box (Figure 3) with an opening mechanism that, if manipulated correctly, released a reward into a feeding tray in front of the box. One string was attached each to the left and the right side of the box. The right side was always functional (this was the right side from the point of view of the subject), and the left side was always non-functional. Subjects gained access to the reward by pulling the string on the right side of the box, which activated a hidden mechanism inside of the box to release the reward. The string on the left side was a non-functional dummy. If a subject pulled the string on the right, half a peanut seed was then released through a tube into the feeding tray at the front of the box. This setup allowed us to see if the observers manipulated the correct string and hence solved the box faster than the control group. Although all subjects were naïve to the test box, some of them had more experience than others from previous experiments with pulling strings (Table 1 [49,50]).

We used two colors of string: red and blue. For both test groups, the red string was always on the right side of the box and was therefore always correct. We did not vary which string was correct for the test groups because the two groups already varied in the identity of the demonstrator, and if the color of the correct string was also different, we would not have been able to disentangle these two variables. Switching the side of the correct string between trials would have theoretically been possible, but not practical, as the strings were securely attached to the box. However, this brought up the potential for subjects to solve the box due to a color preference for one of the strings, as opposed to through social or trial-and-error learning. We therefore ran an additional control (CG2) to look for color preference at the suggestion of an anonymous reviewer, where the blue string was on the right, correct side of the box.

### 2.4. Setup

**Figure 4:**
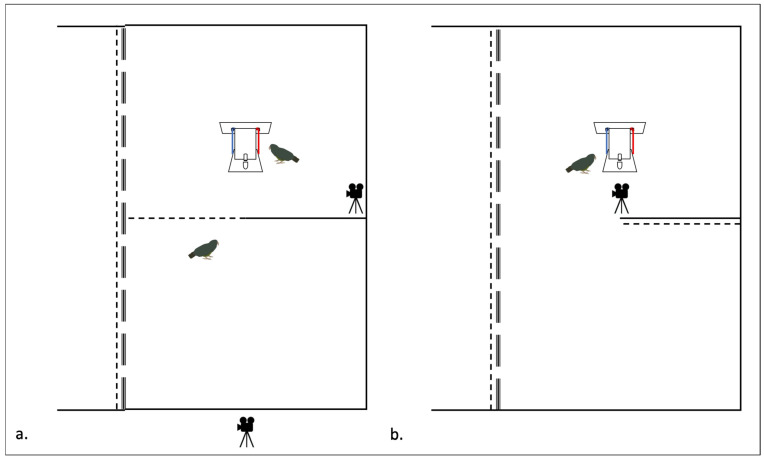
Schematic of social learning tests. (**a**) Demonstration phase, where the test subject is located in the observation compartment while the demonstrator solves the task in the test compartment. A sliding wire-mesh door separates the two compartments while allowing the observer to see the demonstration. (**b**) Test phase, where the demonstrator is gone and the test subject is allowed access to the box for 2 min or until it solves the task, whichever comes first. Functional (red) and non-functional (blue) strings shown.

The apparatus was placed in the middle of the demonstration compartment at a distance of one meter from the closed wire-mesh door. Two cameras recorded each session. One camera was located in the test compartment (Figure 4) and recorded the demonstration and the test sessions at close range. The other was located outside the aviary behind the observation compartment (Figure 4) and recorded the behavior of the observer during the observation session.

To minimize the effect of experimenter presence during the trials, the experimenter strictly avoided eye contact with the subjects and wore sunglasses. A study on African Gray Parrots (*Psittacus erithacus*) showed evidence that the birds were not able to make use of the gaze direction or small gestures of an experimenter [51]. Based on this study result and on the precautions taken, we assume that the presence of the experimenter did not affect the results of the present study in any way.

The procedure of this study was divided into three major steps:

(1) Control group tests (CG1);
(2) Demonstrator training;
(3) Social learning tests (TG1, TG2).

Subjects were assigned to either the two control groups (which included the demonstrators) or the two test groups (see Table 1). As mentioned above, after data collection for control group 1, we trained the two demonstrators taken from that group. Finally, the social learning tests took place.

### 2.5. Control Groups

CG1 consisted of both demonstrators as well as four additional subjects. At this point, the demonstrators had not yet been taught how to solve the task, and so they were still naïve. CG2 consisted of naïve birds that did not participate in any other part of the experiments. The purpose of having two control groups was to test whether there was a color preference for one of the strings (red or blue) over the other. CG1 received the same experimental setup as the test groups, with the red string located on the right, functional side of the box. CG2’s setup differed only in that the blue string was located on the right side of the box, and the red string was on the left, non-functional side.

The control tests comprised three sessions of one trial each, which were carried out in consecutive testing slots (morning and afternoon slots), with a maximum of one day break to avoid extensive interruptions in the testing process. Each trial consisted of a two-minute observation period and subsequent two-minute interaction period with the test-box, for every subject separately.

**Figure 5:**
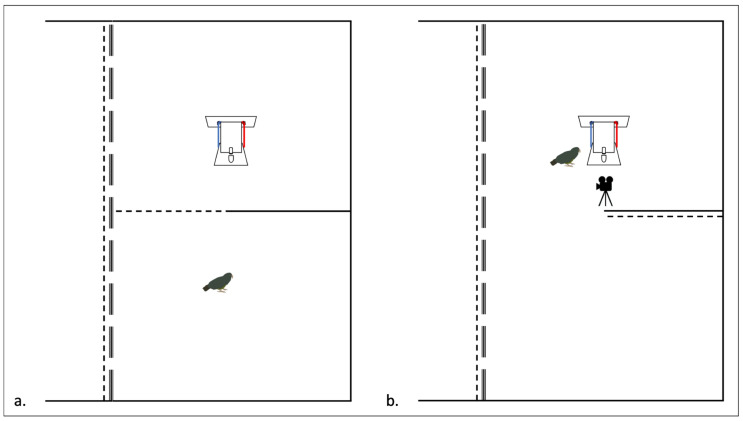
Schematic of control group tests. (**a**) The control subject is given two minutes to observe the box without having access to it. The subject can see the apparatus through the wire-mesh door. (**b**) Test phase, where the control subject is allowed into the test compartment and has a maximum of two minutes to solve the box through trial and error.

Subjects were called into the experimental compartment individually. The subject had two minutes to observe the apparatus through the wire-mesh door (Figure 5a). This observation phase served to match the procedure of the control group tests and social learning tests as closely as possible. Consequently, the control subjects were able to see the test box before interacting with it. The observation period was immediately followed by a test trial. The procedures for the test trials were identical for test and control groups (Figure 4b and Figure 5b).

### 2.6. Demonstrator Training

After they finished participating in the tests for CG1, the two demonstrators An and Sk were trained to open the test box consistently and correctly. Both demonstrators received training sessions of ten trials each, twice a day, until the task was learned. The learning criteria was defined as 100% correct performance in two consecutive sessions. Both demonstrators reached criteria after eight sessions, and they also received refresher sessions directly before the social learning tests.

### 2.7. Social Learning Task

The two test groups were balanced for rank, age, and sex as much as possible (see Table 1). Each group comprised six subjects. Every subject received a total three sessions of one trial each, which were carried out in consecutive testing slots (morning and afternoon slots), with a maximum of a one-day break to avoid extensive interruptions in the testing process. Each session consisted of a demonstration and a test trial, where the subject could interact with the apparatus. As mentioned above, the red string was always on the right, functional side, and the blue string was on the left, non-functional side for both test groups.

#### 2.7.1. Demonstration

First, the observer was called into the observation compartment, where it stayed until the demonstration was complete. Next, the experimenter set up the apparatus. Finally, the demonstrator was called into the test compartment and allowed to solve the box (Figure 4a). After eating its reward, the demonstrator was sent back into the main aviary. The observer was able to view the demonstration from the observation compartment through the wire-mesh door (Figure 4a).

#### 2.7.2. Test Trial

The demonstration was immediately followed by a test trial. Here, the subject was let into the test compartment and had the possibility of interacting with the apparatus (Figure 4b). After a maximum of two minutes, or after successful completion of the task (whichever came first), the trial was ended. The subject was then sent back to the main aviary and the test box was reset. A session was considered invalid if the subject did not touch the apparatus within two minutes. The experimenter timed the test session using a stopwatch. In addition to the video recordings, the experimenter noted the success and failure of every trial manually. Please see Supplementary Materials for video examples of the test.

**Figure 6:**
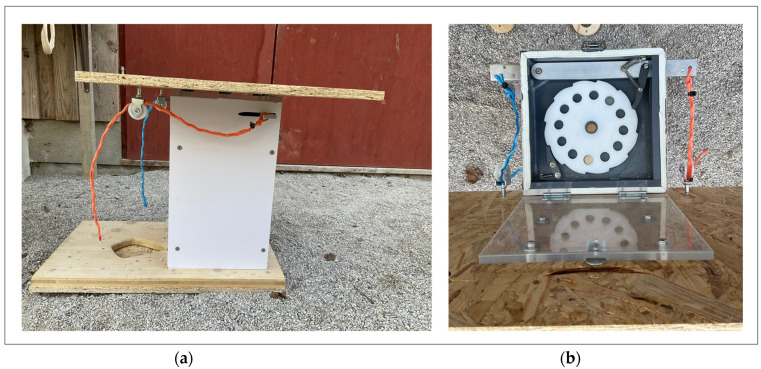
Photographs of the experimental box. (**a**) Side view of the box showing the functional (red) string attached to a lever and threaded through a pulley. When this string is pulled, a small piece of peanut is released into the reward tray at the bottom. (**b**) Top view of box with open lid. By pulling the string, a lever moves and turns the hopper to release a peanut. Only the red string on the right is functional. The blue string is attached to a dummy lever and is non-functional.

Unfortunately, after testing had already begun, we found out that it was possible for the subjects to manipulate the apparatus in such a way as to make it unsolvable. They could do this by pulling the correct string out of the loop, therefore making it impossible to trigger the mechanism (Figure 6). This happened very infrequently (total of 4 out of 54 trials). When it occurred, the experimenter paused the trial, sent the subject back into the observation compartment, reset the device, and allowed the subject to complete the remainder of the trial. All subjects, thus, had the same number of completed trials.

### 2.8. Data Coding and Analysis

#### 2.8.1. Data Coding

The videos of the control group and the social learning test were coded with Solomon Coder [52]. Only the recordings of the test trials (interaction of the subjects with the test-box) were relevant for analysis. All videos were coded in a time resolution of 0.2 s. We coded the test trial duration in seconds, because although the experimenter timed the trial with a stopwatch for 2 min, and the subject was given the command to leave the compartment when the 2 min was up, there could still have been a small amount of variation in the trial length.

We coded the following variables for further analysis: success (subject solved the task and was rewarded) and relative time manipulating the correct string (duration of physical contact with the correct string divided by the total length of the session). All data were summarized in a Microsoft Excel (version 16.59) sheet for further processing (Table S1 data set).

#### 2.8.2. Analysis

To investigate the overall success of solving the apparatus, we compared the number of sessions solved and the number of sessions not solved in model 1. We initially used a model with the response in each individual session and session number as a predictor, However, these suffered from complete separation. We therefore tabulated success as the response in a two-column matrix that included successful and not successful sessions [53]. This response was included in a logistics generalized linear mixed model using a logit-link function, with group and previous experience (coded as “no” or “yes”) as fixed effects. To control for pseudoreplication, we included individual as a random intercept effect. As a test for the fixed effect of group, we conducted a full–null comparison using a null model lacking the fixed effect of group but otherwise being identical in the fixed and random parts of the model.

To investigate whether the proportion of time manipulating the correct string differed between groups, we fitted a generalized linear mixed model with beta error distribution and logit-link function in model 2. As fixed effects, we included group, previous experience and session number. As a random intercept effect, we included individual to avoid pseudoreplication. To avoid overconfident models and to keep the Type I error rate at the nominal level of 0.05 [54,55], we included the slope of session number in individual. Initially, we also included the parameter for the correlation between random intercept and slope, but this was excluded from the model, as this model did not converge. Prior to model fitting, we z-transformed the response to avoid values being exactly zero or one [56]. We also z-transformed session number to a mean of zero and standard deviation of one to ease model convergence and achieve easier interpretable model coefficients [57]. To test the fixed effect of group, we compared the full model to a null model lacking the effect of group, but otherwise being identical in the fixed and random parts of the model.

To investigate if the proportion of time spent manipulating the red strings differed between the two control groups, suggesting color preference, we fitted generalized linear mixed model with beta error distribution and logit-link function. In model 3, we fitted subgroup and session number as fixed effects. Random effects included individual as random intercept effect to avoid pseudoreplication, together with the random slope of session number within individual to avoid overconfident models and to keep Type I error rate at the nominal level of 0.05 [54,55]. The parameter for the correlation between random intercept and slope was excluded from this, as this was not identifiable [58]. Before fitting the model, we transformed the response to avoid values being exactly zero or one [56]. Session number was z-transformed to a mean of zero and standard deviation of one to ease model convergence and achieve more easily interpretable model coefficients [57]. To test the fixed effect of subgroup, we compared the full model to a null model lacking the effect of subgroup, but otherwise being identical in the fixed and random parts of the model.

We fitted the models in R (version 4.2.0 R Core Team): the logistic model 1 was fitted using the function glmer from the lme4 package (Version 1.1.-27 [59]), and beta models were fitted using the function glmmTMB from the glmmTMB package (Version 1.1.5 [60]). All significance levels we report are based on likelihood ratio tests [61]. We calculated confidence intervals for the model estimates of model 1 by applying the function ‘bootMer’ of the package ‘lme4’, using 1000 parametric bootstraps. We obtained confidence intervals of model estimates of model 2 and model 3 by applying the function ‘simulate’ of the package ‘glmmTMB’, using 1000 parametric bootstraps.

For all models, we checked for absence of collinearity by calculating variance inflation factors for models lacking the random effects using the R package “car” version 3.0-12 [62]. These revealed that collinearity was not an issue (max vif: 2.00; 1.04; and 1.00, for model 1, model 2, and model 3, respectively). For each model, we visually inspected the best linear unbiased predictors (BLUPs) of the random effects and confirmed that these were approximately normally distributed [53]. We confirmed that overdispersion was not an issue for any of the models (dispersion parameters: 0.13; 1.02; and 1.12, for model 1, model 2, and model 3, respectively (all *p* > 0.31)). To estimate model stability [63], we excluded the levels of the random effect (individual) one at a time to compare the resulting estimates with those obtained from the model based on all data. Model 2 and model 3 showed good stability. However, model 1 was revealed to have very poor stability. This model instability is caused by complete separation when some individuals are not included in the model, resulting in very large model estimates. We therefore urge a cautious interpretation of the results for model 1.

## 3. Results

### 3.1. Exclusions and Anomalies

In the course of the control group tests, one subject (Ma) had to be excluded due to lack of motivation. All other subjects completed all control sessions. Two test group trials had to be interrupted to reset the box, as the subject deactivated the apparatus by pulling the correct string out of the device (Fr—sessions 2 and 3). That subject was able to finish its test trials with the remaining time. During the video coding, it became evident that the apparatus had been deactivated in two other instances (Pl—session 3, Di—session 1), which the experimenter did not notice at the time of testing. This meant that, after the point the subject deactivated the box, solving the task was no longer possible. To account for this, these sessions were marked as finished at the point when the subject deactivated the box. Consequently, the durations of these trials were shortened (Pl-session 3: 44 s, Di-session 1: 85.8 s). Video coding showed that, excluding the shortened sessions, mean test trial duration was 122.99 s (±3.58 s). When the shortened session durations were included, the mean session duration was 117.18 s (±19.42 s).

### 3.2. Results of the Statistical Analysis

The descriptive statistics showed that only test subjects touched at least one of the two strings in each trial. Test subjects manipulated the correct string in 100% of the trials and the other string in 69.4% of the trials. In the control group, there were three instances (An—session 2 and 3; Fy—session 2) in which the subject, although manipulating other parts of the test box, ignored both of the strings. The subjects in the control group manipulated the correct string in 77.8% of the trials and the other string in 66.7% of the trials.

**Figure 7:**
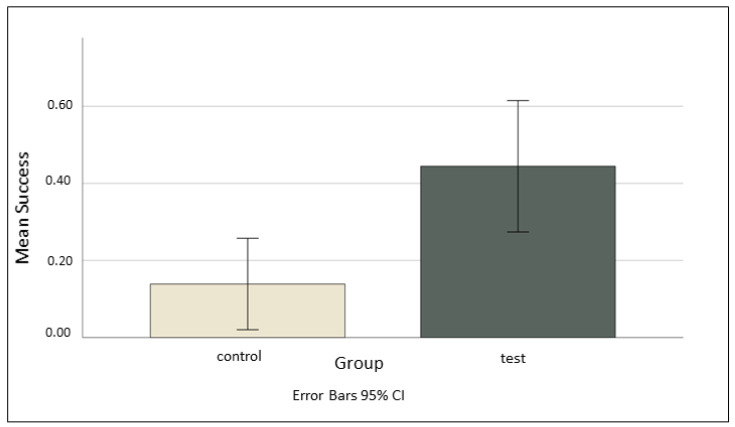
This figure shows the mean number of sessions where the subject was successful in solving the task, organized into control and test groups. The control group was successful in 14% of sessions (5/36), and the test group was successful in 44% of sessions (16/36). There was a non-significant trend towards the test groups being more successful (*p* = 0.056).

We found a strong trend between individuals in the test group and individuals in the control group in overall success of solving the task (model 1, likelihood ratio test comparing full and null model χ^2^(1) = 3.580, *p* = 0.056, with test group subjects solving the task more than control group subjects (Figure 7).

**Figure 8:**
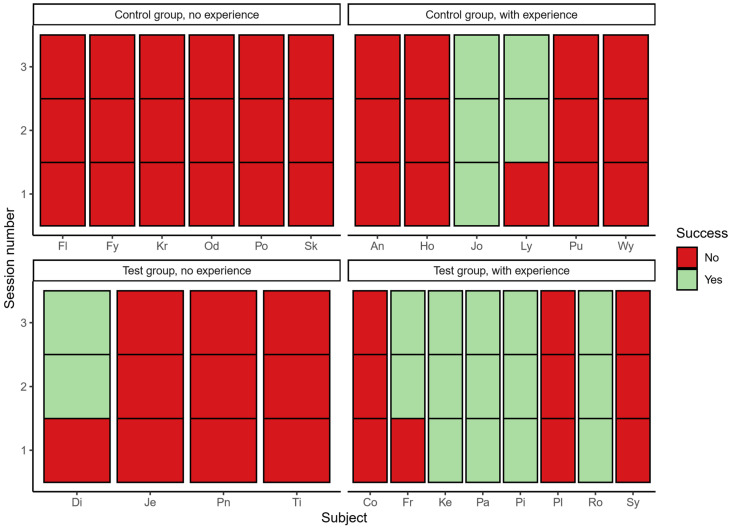
This figure shows each individual subject’s success over all three trials (test or control), organized by whether that subject had past experience with string-pulling. More experienced subjects were successful in solving the task, but not all experienced subjects managed. One inexperienced subject in the test group, Di, solved the task, whereas no inexperienced subjects in the controls group solved the task.

Concerning the effect of previous experience on success, the results are best considered on an individual basis (Figure 8). Of the 12 subjects in the control group, 6 had previous experience in string-pulling tasks, and 6 had none. Of these, two experienced individuals solved the task (Jo 3/3 sessions; Ly 2/3 sessions). No other subjects in the control group solved the task, including the remaining four with previous experience (An, Ho, Pu, Wy, see Table 1). Of the 12 subjects in the test group, 8 had previous experience and 4 did not. Here, a total of six subjects solved the task: one out of four with no previous experience solved the task (Di, 2/3 sessions), and five out of eight subjects with previous experience solved the task (Fr, 2/3 sessions; Ke, Pa, Pi, Ro 3/3 sessions).

**Figure 9:**
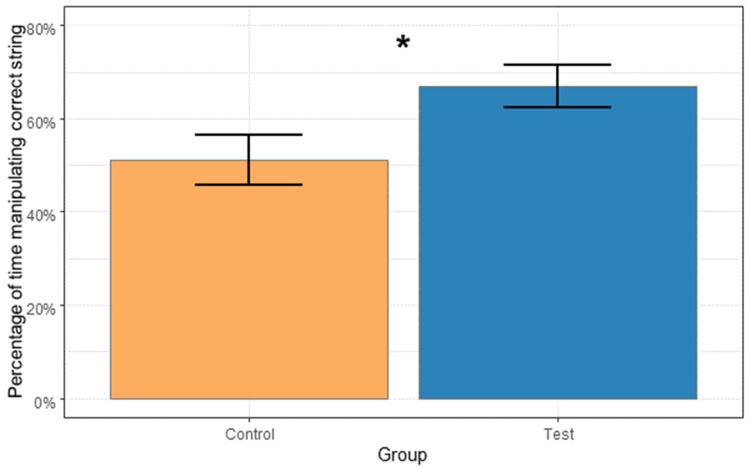
This figure shows the mean proportion of time that the subjects spent manipulating the correct string during a trial, compared to the total string manipulation time, organized into control and test groups. The control group manipulated the correct string in a proportion of 0.51 of the time, and the test group manipulated the correct string significantly more, in a proportion of 0.67 of the time (* *p* = 0.029).

Overall, there was an effect of group (model 2, likelihood ratio test comparing full and null model χ^2^(1) = 4.742, *p* = 0.029, Figure 9) on proportion of time spent manipulating the correct string compared to the total time manipulating both strings. Individuals in the control group manipulated the correct string in a proportion of 0.51 of the time, while individuals in the test group manipulated the correct string in a proportion of 0.67 of the time, independent of previous experience.

We did not find a difference between subgroups in proportion of time manipulating the red string compared to the blue string (model 3, likelihood ratio test comparing full and null model χ^2^(1) = 0.012, *p* = 0.911). Individuals in subgroup CG1 manipulated the red string in a proportion of 0.605 of the time, compared to a proportion of 0.594 of the time for individuals in subgroup CG2.

## 4. Discussion

The Kea that had the opportunity to observe a trained conspecific solve the task showed a trend towards higher success in completing the task, suggesting that social learning occurred. Moreover, the test groups spent significantly more time manipulating the correct string compared the control groups. This further supports that perceptual factors, i.e., stimulus and/or local enhancement, were utilized by the observers, when it was necessary to solve the task, in contrast to the previous study where it was not [43]. Alongside the statistical analysis, the experimenter subjectively observed that the unsuccessful control subjects increasingly lost interest in the apparatus and engaged in other activities in the experimental compartment as sessions progressed. In contrast, the test subjects displayed explorative behavior towards the test box throughout all of the three test trials. Observing a conspecific performing actions at the test box resulted in test subjects interacting with the test box longer than controls.

Our current findings support those of a previous study [42], namely that Kea benefit from social information through perceptual factors, and that it leads to greater persistence. Huber and colleagues [42] showed that observing skilled conspecifics open three sets of locking devices on a food container resulted in longer and more sustained exploratory behavior in the observers compared to a control group. Huber et al. hypothesized that stimulus enhancement was one of the responsible learning mechanisms, although local enhancement could also have played a part, as the locks were all situated next to one another on the box. Additionally, motivational shifts towards species-typical movements may have been triggered by the demonstration [42]. Our results suggest that the salient actions of the demonstrator may have made the correct string of the locked test box more attractive to the observers, leading to more explorative behavior as well as prolonged manipulation of this stimulus. Stimulus enhancement plays an important role in behavior acquisition in other bird species, like Common Ravens (*Corvus corax*) [27], Eurasian Jackdaws (*Corvus monedula*) [64], and Greylag Geese (*Anser anser*) [65]. Even relatively asocial birds like Eurasian Jays (*Garrulus glandarius*), which do not live in social groups, show indications of stimulus enhancement after observing a conspecific accomplishing a task [66]. Nonetheless, the level of gregariousness could be a deciding factor in the transfer of information in a social learning task, as domesticated fowl (*Gallus domesticus*), selectively bred to live in larger and more dynamic social groups, were found to solve a puzzle-box lid-opening task faster than the ancestral Red Jungle Fowl (*Gallus gallus*) with faster approaches and increased pecking of the relevant lid [67].

Observing the whole sequence of a behavior may be important for subsequently directing more sustained actions towards a stimulus. This could be the deciding difference even if all subjects, observers as well as controls, are interested in exploring the presented stimulus [42,68]. Correspondingly, our results show that although all subjects were interested in manipulating the apparatus, control subjects ignored both of the strings in some trials. On the other hand, test subjects touched the correct string in every trial, and showed sustained actions toward the correct string and manipulated it for a significantly longer period of time than the control group.

However, while perceptual factors can direct observers’ attention towards a particular stimulus or location [15,17,21,22], other mechanisms of social learning may have been responsible for the observers’ higher success in gaining access to the food reward. Stimulus enhancement, as the name already suggests, enhances the behavior towards a stimulus, but that does not entail learning any specific action [22]. It affects the likelihood of learning, as the individuals engage with the correct stimulus, but it does not constitute the associative learning mechanism [65], which would be responsible for understanding how the mechanism of the locked food container works. In our study, test subjects were more effective in performing the task than the control group, albeit non-significantly. Six out of twelve test subjects managed to solve the task and obtain the reward, while only two control subjects successfully solved the task. These results indicate the ability of Kea to socially acquire information about purposeful behavior from their conspecifics.

Prior studies already suggest that Kea use social information in learning processes [34,35,43]. It is hypothesized that observers learned something about the affordances of social learning tasks, suggesting emulation processes as effective learning mechanisms [42]. In the present experiment, the Kea may have learned about the function of the string by observing their conspecific manipulating it. Nonetheless, as there was no alternative way to solve the task than the one observed (pulling on the string), emulation is not a possible explanation here. There is also evidence for Kea rapidly neglecting social information in favor of overt exploration [35]. This would suggest autonomous associative learning following stimulus and/or local enhancement in our study. As the action itself was within the natural range of behaviors for our subjects, further analyses and refined methodologies are necessary to specify and differentiate alternative mechanisms.

It should be noted that more than half (14 out of 24) subjects had previous experience with some sort of string-pulling task. The successful subjects, with one exception, all had such previous experience; thus, this could have provided these birds with important knowledge to apply to the current setup. However, due to the nature of working with a lab population of long-lived subjects, there was also a correlation between age and experience in string-pulling tasks that could not be disentangled. Age correlates with test experience in general in this group of Kea, not just string-pulling, and almost all subjects that solved the task were older. Due to experience, older birds might have paid more attention to the demonstration. Conversely, while experience was clearly a supporting factor, not all birds with previous experience successfully completed the task. Additionally, test subjects manipulated the correct string for longer than controls regardless of experience, and two of the three trials where a subject did not interact with a string at all were performed by an experienced bird (Anu). For these reasons, we argue that the effect of a demonstration on subjects’ behavior should be seen as independent of previous experimental experience.

As the manipulation of the correct, or demonstrated, string was significantly higher in test birds than control birds, stimulus enhancement stands as the likely underlying mechanism. However, while there was only one apparatus, the size of the setup meant that even positioning equidistant between both strings still required the subject to orient their body towards one or the other to manipulate it. It is unclear if this could have led to local enhancement effects also playing a part in the test birds’ success. Even if this were the case, the design of the box did not allow for the functional string to be located on both sides, meaning that a counterbalancing of correct string was not possible. This short-coming in design should be addressed in future setups to be able to disentangle stimulus and local enhancement.

Overall, the present study provides further evidence that Kea are capable of social learning. In contrast to a recent study with the same group using a two-action task methodology [43], effects of perceptual factors were found to be significant using the current simplified methodology. Diamond and Bond [31] suggest that learning in Kea is determined by two main processes: play behavior and social facilitation. Opportunism, social foraging, iteroparity, and extended association with the parents seem to favor social learning in animals [38,69,70]. Kea fulfill many of these conditions [31] and therefore represent a species in which the mechanisms and function of social learning are worth investigating.

## 5. Conclusions

In this study, we used a simple methodology to test for aspects of social learning in captive Kea parrots. Test subjects viewed a demonstration of a conspecific pulling the correct of two possible strings, which was attached to a box and released a food reward. Test subjects were then given access to the box to try and solve it for themselves. Control subjects were given access to the box to solve without viewing a demonstration beforehand. Our study showed that test subjects manipulated the correct string for significantly longer than controls, and there was a trend towards test subjects being more successful in solving the task—six out of twelve test subjects solved the task successfully, but only two out of twelve control subjects managed this. Previous experience in string-pulling tasks increased the chance that a subject would solve the task, but was neither necessary nor sufficient for success. In contrast to a recent previous study on social learning in this group of captive Kea, we were able to show that perceptual factors played a clear role in social learning. Having established that, when required to solve a task, Kea do attend to perceptual factors, future research will focus on further disentangling stimulus and local enhancement effects.

## Figures and Tables

**Table 1:** Subject information.

Subject	Group *	Age	Sex	Previous Experience
An	CG1	15	M	yes
Fy	CG1	6	F	no
Jo	CG1	23	M	yes
Od	CG1	7	M	no
Pu	CG1	9	F	yes
Sk	CG1	5	M	no
Fl	CG2	1	F	no
Ho	CG2	16	F	yes
Kr	CG2	8	F	no
Ly	CG2	16	F	yes
Po	CG2	1	M	no
Wy	CG2	16	F	yes
Ke	TG1	18	M	yes
Pa	TG1	12	M	yes
Pi	TG1	18	M	yes
Pl	TG1	15	F	yes
Pn	TG1	5	M	no
Sy	TG1	15	F	yes
Co	TG2	15	F	yes
Di	TG2	5	F	no
Fr	TG2	18	M	yes
Je	TG2	7	M	no
Ro	TG2	14	M	yes
Ti	TG2	4	F	no

* CG1—control group 1, CG2—control group 2, TG1—test group 1, TG2—test group 2; F—female, M—male.

## Data Availability

The original data set for this study is available in the Supplementary Materials.

## Supplementary Materials

The following supporting information can be downloaded at: https://www.mdpi.com/article/10.3390/ani14111651/s1, Table S1: Data Set, Table S2: Subject previous experience; Video S1: Demonstration Trial, Video S2: Test Trial.
